# Alcohol use and viral suppression in HIV-positive Kenyan female sex workers on antiretroviral therapy

**DOI:** 10.1371/journal.pone.0242817

**Published:** 2020-11-24

**Authors:** Jessica E. Long, Barbra A. Richardson, George Wanje, Kate S. Wilson, Juma Shafi, Kishorchandra Mandaliya, Jane M. Simoni, John Kinuthia, Walter Jaoko, R. Scott McClelland

**Affiliations:** 1 Department of Epidemiology, University of Washington, Seattle, Washington, United States of America; 2 Department of Global Health, University of Washington, Seattle, Washington, United States of America; 3 Department of Biostatistics, University of Washington, Seattle, Washington, United States of America; 4 Department of Medicine, University of Washington, Seattle, Washington, United States of America; 5 Department of Medical Microbiology, University of Nairobi, Nairobi, Kenya; 6 Department of Psychology, University of Washington, Seattle, Washington, United States of America; 7 Kenyatta National Hospital, Nairobi, Kenya; University of the Witwatersrand, SOUTH AFRICA

## Abstract

**Background:**

Excessive alcohol intake has been associated with poor adherence to antiretroviral therapy (ART). The impact of alcohol on viral suppression is particularly important among groups at high risk of HIV transmission, such as female sex workers (FSWs). Few studies have directly evaluated the association between alcohol use and HIV viral load. We hypothesized that hazardous or harmful alcohol use is associated with detectable plasma viral load among HIV-positive FSWs.

**Methods:**

A prospective cohort study was conducted among HIV-positive FSWs in Mombasa, Kenya. Hazardous or harmful alcohol use was assessed yearly and defined as an Alcohol Use Disorders Identification Test (AUDIT) score ≥7. Detectable viral load was assessed every six months and defined as ≥180 c/mL. Adherence measures were collected monthly and included late ART refill (>48 hours) and self-reported adherence, using both a validated self-rating scale of ability to take medication and visual analog scale (VAS) of ART use in the last month. Generalized estimating equations were used to estimate adjusted relative risks (aRR) and 95% confidence intervals (CI).

**Results:**

This analysis included 366 participants followed monthly between October 2012 and March 2018. At baseline, AUDIT scores indicated hazardous alcohol use (AUDIT 7–15) in 14.3%, harmful alcohol use (AUDIT 16–19) in 1.4%, and alcohol dependency (AUDIT ≥20) in 1.4% of participants. After adjusting for potential confounders, a combined exposure including hazardous, harmful, and dependent alcohol use was not associated with detectable viral load (aRR 1.10, 95%CI 0.63–1.92) or late ART refill (aRR 1.13, 95%CI 0.82–1.56), but was associated with lower self-rated ability to take medication (aRR 2.38, 95%CI 1.42–3.99) and a lower rate of self-reported perfect ART adherence by VAS (aRR 2.62, 95%CI 1.84–3.71).

**Conclusions:**

In this FSW cohort, while participants reporting hazardous, harmful, or dependent alcohol use were not more likely to have a detectable viral load, they were more likely to report lower ART adherence. These results suggest that interventions targeting alcohol use among this population of FSWs may not have a large impact on viral suppression.

## Introduction

Suppression of HIV viral replication is critical to maintain health and prevent HIV transmission. Viral suppression requires adherence to daily antiretroviral therapy (ART), which can be difficult due to multiple psychosocial and structural barriers such as fear of disclosure of HIV status, stigma, and difficulty in following a daily regimen [1]. Studies in high- and low-income countries have demonstrated that alcohol use is associated with poor ART adherence [1–4]. Fewer studies have examined the association between alcohol use and viral suppression, particularly in low-income settings, and these provide conflicting results [5–11]. With modern ART regimens, adherence >80% is sufficient to maintain viral suppression [12]. In this context, it is important to examine not only the association between alcohol use and adherence, but also the relationship between alcohol and viral suppression.

Female sex workers (FSWs) in sub-Saharan Africa are disproportionately affected by both HIV and alcohol use [7]. Based on data from the National AIDS and STI Control Programme, 29% of FSWs in Kenya are living with HIV [13]. In addition, HIV-seropositive FSWs face greater barriers to ART adherence compared to those who don’t sell sex, with evidence suggesting that ART adherence is lower among FSWs than non-sex workers in sub-Saharan Africa [7, 14–16]. A systematic review and meta-analysis reported the prevalence of current use of ART was 23% among FSWs living with HIV in Kenya [15]. Sex work is often conducted in bars and nightclubs, and FSWs may use alcohol to cope with the stress of sex work [7, 17]. Understanding and addressing barriers to ART adherence in this key population is important for achieving the 95-95-95 target of having 95% of those on ART virally suppressed by 2030 [18].

This prospective cohort study tested the hypothesis that alcohol use is associated with a lower likelihood of viral suppression in FSWs receiving ART.

## Methods

### Population and procedures

Data were collected from HIV-positive FSWs in Mombasa, Kenya. Detailed methods have been published [19]. Briefly, women were recruited using community outreach in FSW workplaces. Eligibility criteria included age ≥16 years, laboratory confirmed HIV infection, receiving ART, and reporting exchanging sex for money or in-kind payment.

All participants provided written informed consent, then completed a baseline standardized face-to-face interview. Self-reported data were collected on socio-demographics, reproductive and sexual health, and history of violence. Depression was assessed using the Patient Health Questionnaire-9 (PHQ-9) [18]. Blood samples were collected for viral load testing and CD4 counts. A physical examination including pelvic examination with sample collection for STI testing was performed.

Alcohol use was measured at baseline through the Alcohol Use Disorder Identification Test (AUDIT), an internationally validated tool to screen for alcohol use in the prior 12 months [20, 21]. Among women, AUDIT scores of 0–6 indicate abstinence or non-hazardous alcohol use, 7–15 hazardous alcohol use (drinking that increases risk of future harm), 16–19 harmful alcohol use (drinking that impairs physical, mental, or social health), and 20–40 alcohol dependency [21]. In this population, few women had AUDIT scores in the harmful or dependent range, so scores were dichotomized, comparing scores of 0–6 to scores ≥7. Antiretroviral therapy adherence was evaluated through three measures. First, self-reported ability to take ART was assessed using a validated 5-point self-rating scale of excellent, good, fair, poor, or very poor [22]. For analysis, this was transformed to a binary score of “excellent” or “less than excellent”. Second, women were asked to mark a visual analog scale (VAS) representing the percent of ART doses taken in the past month. In this population, the majority of VAS responses indicated 100% of doses taken, so this variable was dicohtomized to compare 100% versus <100% adherence [23, 24]. Third, pharmacy data were used to identify late ART refills, defined as refills occurring >48 hours after medications from the prior refill would have run out, assuming perfect adherence [25].

Women were asked to return for monthly follow-up visits to collect data on ART adherence. Every three months CD4 counts were repeated. Participants completed the PHQ-9 and viral load testing every six months. AUDIT score and history of violence were assessed annually. Participants were compensated 250 Kenyan shillings (approximately USD $2.50) for travel at each visit. Ethics approval was obtained from the Kenyatta National Hospital Ethics and Research Committee and the University of Washington Human Subjects Research Committee.

### Laboratory procedures

Detectable plasma viral load was measured as plasma HIV RNA ≥180 copies/milliliter (Hologic, San Diego, CA). This cut point was higher than the lower limit of detection for the assay (<30 c/mL) because some 100 mL samples had to be diluted 6-fold, to a final volume of 600 mL before testing. Only viral loads collected after ≥3 months of ART use were included [26]. Testing for STIs was performed using the Hologic Aptima detection system (Hologic, San Diego, CA). Vaginal swabs were tested for prostate-specific antigen (PSA) using ABAcard p30 (Abacus Diagnostics, West Hills, CA). Positive PSA indicates semen exposure in the past 24–48 hours [27]. The BD FACSCount (BD Biosciences, San Jose, CA) was used to measure CD4^+^ T-cell count.

### Statistical analysis

The AUDIT was administered annually, and scores were carried forward for analyses until the next assessment. To account for repeated measures from individual participants, associations between AUDIT score ≥7 and viral suppression (primary outcome) and ART adherence (secondary outcomes) were evaluated using generalized estimated equations with log link, Poisson family distribution, independence working correlation structure, and robust standard errors. After estimating unadjusted associations, a multivariable model was built using a forward manual selection process. All models were adjusted for age based on consistent prior findings of associations with alcohol use and adherence [28, 29]. Additional covariates including PHQ-9 score, intimate partner violence, and sexual behavior, were selected as potential confounders based on published associations [30–32]. These variables were modelled as time-varying in analyses. Variables were retained if they shifted the effect estimate for the association between AUDIT score ≥7 and the outcome by ≥10%. Sensitivity analyses were conducted to fully fit each model with all covariates found to be associated with the outcome examined in that model.

Collinearity between AUDIT score ≥7 and additional variables was evaluated by calculating change in the standard error of the model when each variable was added. When collinearity was suspected (>10% change in standard error when variable is added to the model), correlation between the variables was evaluated using Spearman’s rank correlation coefficient; if a variable was highly correlated (r ≥0.6) it would be removed from analysis. To assess whether the effect of alcohol on viral suppression differed by age group, regression analyses were conducted including an interaction term between AUDIT score ≥7 and age category (20–29, 30–39, 40–49, and ≥50 years of age), then were repeated stratifying by age. All analyses were conducted in Stata version 15.1 (College Station, TX, USA, 2017).

## Results

### Sample characteristics

There were 481 FSWs enrolled between October 2012 and April 2017, with follow-up through March 2018. Of the enrolled women, 115 were excluded from this analysis. Seventy had no plasma HIV RNA result after >3 months on ART, 44 were not on ART, and one had no AUDIT score. The remaining 366 participants contributed a total of 11,482 visits (1,131 person-years of follow-up). The median number of visits per participant was 48 (interquartile range [IQR] 39–59).

Baseline characteristics are presented in Table 1. The median age was 40 years (IQR 34, 44). Half the cohort reported a regular partner who was not a client in the prior three months (N = 191, 52.3%). A quarter of participants reported either mild (N = 67, 18.3%) or moderate to severe (N = 30, 8.2%) depressive symptoms. Intimate partner violence in the previous 12 months was reported by 20.2% (N = 74). AUDIT scores at baseline indicated hazardous alcohol use (score 7–15) in 14.3% (N = 52) of participants, harmful use (16–19) in 1.4% (N = 5), and alcohol dependency in 1.4% (N = 5). Of the 207 women who had been prescribed ART for at least three months and had baseline viral load measured, 15.9% (N = 33) were detectable.

**Table 1:** Characteristics of 366 study participants at enrollment.

Characteristic	N	Median (IQR) or n (%)
**Age**	366	40 (34, 44)
**Highest education level ≥ 8 years**	366	210 (57.4)
**Marital Status**	366	
Never married		86 (23.5)
Currently married		27 (7.4)
Widowed/divorced		253 (69.1)
**Workplace**	366	
Bar/restaurant		224 (61.2)
Nightclub		81 (22.1)
Home/other		61 (16.7)
**Years since first sex work**	366	
Less than 5		63 (17.2)
5 to 9		113 (30.9)
10 or more		190 (51.9)
**Partner factors**		
Regular partner in past 3 months	365	191 (52.3)
Casual partner in past 3 months	366	152 (41.5)
**Contraceptive use**	366	
None		132 (36.1)
Condoms only		117 (32.0)
Short / medium acting (DMPA, OCP)		63 (17.2)
Long acting (Implants, TL, IUCD, hysterectomy)		54 (14.8)
**Self-reported sexual behavior in the past 7 days**		
Condomless sex	366	38 (10.4)
Abstinent in past week	366	153 (41.8)
**Subset of women not abstinent in past week (n = 213)**:		
100% condom use in past week	213	175 (82.2)
Number of sex acts in past week	213	2 (1, 3)
Number of sex partners in past week	213	1 (1, 3)
**Depressive symptoms by PHQ-9**	366	
Minimal (0 to 4)		269 (73.5)
Mild (5 to 9)		67 (18.3)
Moderate / severe (10 or higher)		30 (8.2)
**Alcohol use by AUDIT score**	365	
No or non-hazardous alcohol use (<7)		303 (83.0)
Hazardous alcohol use (7–15)		52 (14.3)
Harmful alcohol use (16–19)		5 (1.4)
Alcohol dependency (20–40)		5 (1.4)
**HIV disclosure**	365	
Not disclosed to anyone outside healthcare setting		108 (29.6)
Disclosed to someone but not regular partner^a^		172 (47.1)
Disclosed to regular partner		85 (23.3)
**History of violence and controlling behaviors**		
Any sexual abuse in the past 12 months	366	24 (6.6)
Any physical abuse in the past 12 months	366	24 (6.6)
Controlling behaviors by a regular partner	366	168 (45.9)
Intimate partner violence in the past 12 months	366	74 (20.2)
**Clinical and biomarkers**		
Baseline CD4+ count (cells/mm^3^)	366	471.5 (296, 604)
Chlamydia infection	361	3 (0.8)
Gonorrhea infection	361	12 (3.3)
Trichomonas infection	366	9 (2.5)
Semen detection by PSA test	366	61 (16.7)

^a^Disclosure to someone could include family, friends, religious leaders, etc. Regular partner is defined as husband, boyfriend, or other regular partner who is not a client.

### Alcohol use and detectable viral load

AUDIT score was collected at 1,066 visits, of which 100 (9.4%) were scores ≥7. A total of 1,925 visits included viral load testing after ≥3 months on ART. Of these, 291 (15.1%) had a detectable viral load. In univariate analyses, AUDIT score ≥7 was associated with a 1.59-fold higher relative risk of having a detectable viral load (95% confidence interval [CI] 0.93–2.69) compared to AUDIT score <7 (p = 0.084) (Table 2). This association was attenuated when adjusted for age (adjusted relative risk [aRR] 1.10, 95%CI 0.63–1.92, p = 0.74). Other variables, including PHQ-9 score, history of intimate partner violence, and indicators of sexual behavior had statistically significant associations with AUDIT score ≥7 but were not retained in the final multivariable model after stepwise regression was performed.

**Table 2:** Univariate and multivariate risk ratios (RR) and 95% confidence intervals (CI) for the association of hazardous/harmful/dependent alcohol use, as measured by AUDIT score ≥ 7, on viral suppression and self-reported and objective measures of ART adherence.

Outcome	AUDIT Score		Univariate regression estimates		Multivariate regression estimates	
	<7	≥7	Risk Ratio (95%CI)	p-value	Adjusted Risk Ratio (95%CI)	p-value
**Viral load**						
Undetectable	1,491 (85.3%)	142 (81.1%)	REFERENCE		REFERENCE	
Detectable	258 (14.8%)	33 (18.9%)	1.59 (0.93, 2.69)	0.084	1.10 (0.63, 1.92)^a^	0.739
**ART refill**						
Timely	7,043 (80.1%)	736 (77.5%)	REFERENCE		REFERENCE	
Late (>48 hours)	1,751 (19.9%)	214 (22.5%)	1.38 (0.99, 1.93)	0.056	1.13 (0.82, 1.56) ^a^	0.442
**Self-rated ability to take ART**						
Excellent	8,530 (96.8%)	872 (90.6%)	REFERENCE		REFERENCE	
Less than excellent	280 (3.2%)	91 (9.5%)	3.68 (2.48, 5.44)	<0.001	2.41 (1.48, 3.92) ^b^	<0.001
**Self-rated ART adherence**						
Complete (100%)	8,604 (94.9%)	863 (85.8%)	REFERENCE		REFERENCE	
Incomplete (<100%)	467 (5.2%)	143 (14.2%)	3.44 (2.44, 4.85)	<0.001	2.40 (1.71, 3.38) ^a^	<0.001

^a^Adjusted for age (continuous).

^b^Adjusted for age (continuous) and number of sex acts in the last week.

### Alcohol use and adherence

Participants refilled ART at 9,751 visits, of which 1,968 (20.2%) were refilled late. In univariate analysis, AUDIT score ≥7 was associated with late refill (RR 1.38, 95%CI 0.99–1.93, p = 0.056). This association was attenuated in the multivariable model adjusted for age (aRR 1.13, 95%CI 0.82–1.56, p = 0.442).

Participants completed the ART adherence self-rating scale at 9,780 visits, during which 371 (3.2%) indicated less than excellent perceived ability to take ART. In univariate analysis, compared to visits with AUDIT score <7, AUDIT score ≥7 was significantly associated with a 3.68-fold (95%CI 2.48–5.44) higher relative risk of reporting less than excellent ability to take ART. This association was somewhat attenuated (aRR 2.42, 95%CI 1.48–3.92, p<0.001) but remained highly significant in adjusted analyses that included age and number of sex acts in the last week (a proxy for current level of engagement in sex work).

The VAS of ART adherence was conducted at 10,085 visits, of which 613 (6.1%) indicated <100% adherence. AUDIT score ≥7 was significantly associated with a 3.44-fold (95%CI 2.44–4.85, p<0.001) higher risk of reporting <100% adherence. Similar to the self-rating scale, this association was attenuated (aRR 2.41, 95%CI 1.71–3.38, p<0.001) but remained highly significant with adjustment for age. For all outcomes, sensitivity analyses that fully fit each model with all variables associated with the outcome did not substantially change the inference of the results. These data are provided as S1 File.

### Age and alcohol use

**Fig 1:**
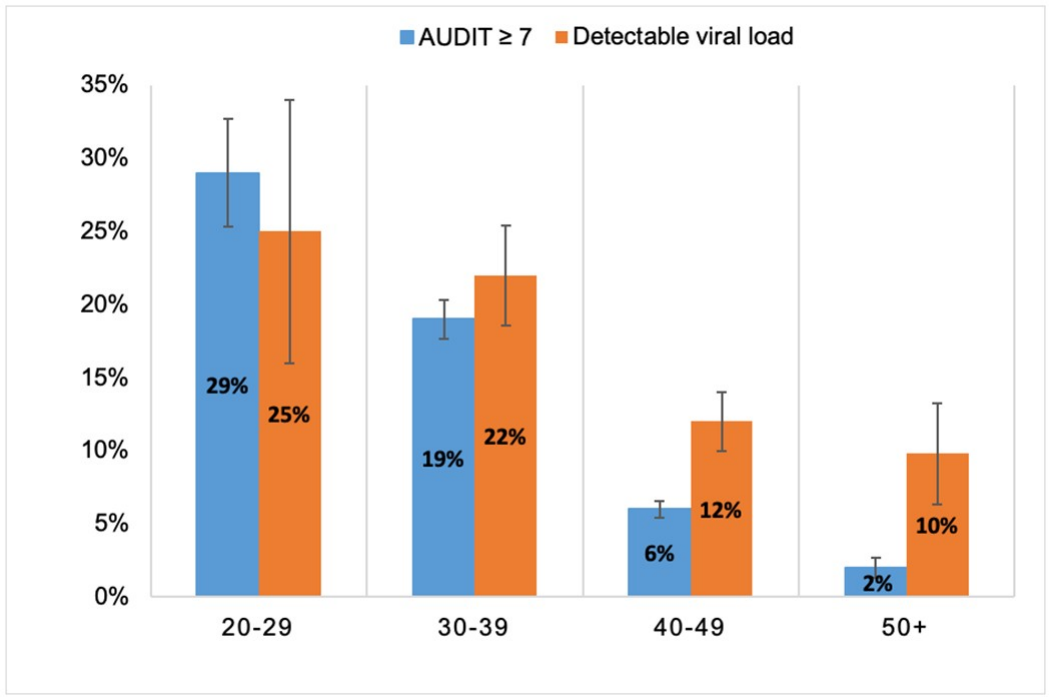
Percent of study visits with AUDIT score ≥7 and detectable viral load by age group. This figure displays the percent of all study visits in which the AUDIT score was ≥7 and viral load was detectable (plasma HIV RNA load ≥180 copies/milliliter), stratified by age group. During the analysis period, 579 study visits were conducted with participants age 20–29 years, 3,387 with those age 30–39, 5,722 with those age 40–49, and 1,794 with those age 50–61 years.

Age was the only variable for which there was evidence of collinearity with alcohol use, resulting in a >10% increase in the standard error when added to each model. However, the correlation between age and AUDIT score was weak (Spearman’s rank correlation coefficient ρ = -0.26, p-value <0.001), so it was retained in the primary models. Given the large attenuating effect of age on the association between alcohol use and detectable viral load in these analyses, as well as this evidence of collinearity, the association between AUDIT score ≥7 and detectable viral load was further evaluated through stratification by age group. The percentage of visits with AUDIT score ≥7 and detectable viral load decreased dramatically with increasing age (Fig 1). No statistically significant evidence of effect modification was found when examining interaction between AUDIT score ≥7 and age category, suggesting that the relationship between AUDIT score and detectable viral load did not significantly differ by age group (all p-values for interaction ≥0.10).

To further explore the effects of age, the primary analyses were repeated without inclusion of age as a covariate, and instead stratifying by age. Due to sparse data in age categories, age was dichotomized at the median (age 42 years). These results did not differ substantially from the primary findings (Table 3). An AUDIT score ≥7 was not significantly associated with detectable viral load in either age group (Age 20–41: RR 1.12, 95%CI 0.61–2.08, p = 0.706; Age 42–61: RR 1.61 95%CI 0.58–4.46, p = 0.361).

**Table 3:** Risk ratios (RR) and 95% confidence intervals (CI) for the association between hazardous/harmful/dependent alcohol use, viral suppression, and ART adherence measures, stratified by age above and below the median (42 years).

**Age 20–41**				
**Outcome**	**AUDIT Score**		**Regression estimates**	
	**<7**	**≥7**	Risk Ratio (95%CI)	p-value
**Viral load**				
Undetectable	598 (79.6%)	110 (80.3%)	REFERENCE	
Detectable	153 (20.4%)	27 (19.7%)	1.12 (0.61, 2.08)	0.706
**ART refill**				
Timely	2,894 (79.1%)	562 (75.7%)	REFERENCE	
Late (>48 hours)	763 (20.9%)	180 (24.3%)	1.32 (0.92, 1.91)	0.134
**Self-rated ability to take ART**				
Excellent	3,539 (96.0%)	679 (90.1%)	REFERENCE	
Less than excellent	148 (4.0%)	75 (10.0%)	2.30 (1.31, 4.03) ^†^	0.004
**Self-rated ART adherence**				
Complete (100%)	3,539 (93.1%)	674 (85.0%)	REFERENCE	
Incomplete (<100%)	264 (6.9%)	119 (15.0%)	2.50 (1.66, 3.77)	<0.001
**Age 42–61**				
**Outcome**	**AUDIT Score**		**Regression estimates**	
	**<7**	**≥7**	Risk Ratio (95%CI)	p-value
**Viral load**				
Undetectable	893 (89.5%)	32 (84.2%)	REFERENCE	
Detectable	105 (10.5%)	6 (15.8%)	1.61 (0.58, 4.46)	0.361
**ART refill**				
Timely	4,149 (80.8%)	174 (83.7%)	REFERENCE	
Late (>48 hours)	988 (19.2%)	34 (16.4%)	0.92 (0.43, 1.96)	0.833
**Self-rated ability to take ART**				
Excellent	4,991 (97.4%)	193 (92.3%)	REFERENCE	
Less than excellent	132 (2.6%)	16 (7.7%)	2.73 (0.66, 11.24)^a^	0.164
**Self-rated ART adherence**				
Complete (100%)	5,065 (96.2%)	189 (88.7%)	REFERENCE	
Incomplete (<100%)	203 (3.9%)	24 (11.3%)	3.20 (1.35, 7.60)	0.008

^a^Adjusted for number of sex acts in last week.

## Discussion

In this longitudinal study of HIV-seropositive FSWs receiving ART, the overall prevalence of hazardous, harmful, or dependent alcohol use was low and was not associated with having a detectable viral load. Both alcohol use and detectable viral load decreased with older age, but the relationship between alcohol use and viral load was similar across age groups. Interestingly, while self-report of lower adherence using two validated methods was uncommon, women with hazardous, harmful, or dependent alcohol use were significantly more likely to report lower ART adherence using both a validated adherence self-rating scale and VAS.

The prevalence of hazardous, harmful, or dependent alcohol use, measured through AUDIT score, was lower in this cohort than in other cohorts of FSWs, both in Kenya [33] and in various parts of Asia and Latin America [34–36]. The effect of alcohol use on self-reported ART adherence in this study is consistent with previous research in other populations. A recent systematic review and meta-analysis of studies in sub-Saharan Africa, including male and female populations, found a consistent association between alcohol use and varied measures of lower ART adherence or ART non-use, with a pooled effect estimate of 2.25 (95%CI 1.87–2.69) [2]. The review identified only one study conducted among FSWs [7]. In this cross-sectional study from Malawi, non-use of ART was 1.9-fold higher (95%CI 1.0–3.8) in FSWs with AUDIT score ≥16 (harmful or dependent alcohol use) compared to those with scores <16.

The association between alcohol use and detectable viral load has been examined in a number of studies in high-income settings, with mixed results [37–42]. Fewer studies have examined this association in sub-Saharan Africa, and both exposure definitions and results have varied in differing contexts. The only study of African FSWs, conducted in Malawi, found no significant association between harmful or dependent alcohol use and detectable viral load (aPR 1.7, 95%CI 0.5–5.5) [7]. Studies of general population clinic attendees in South Africa, Lesotho, and Morocco also found no association between alcohol use and detectable viral load [6–9]. In contrast, alcohol consumption was associated with detectable viral load among those on ART in a clinic-based cohort study in Botswana (aOR 1.7, 95%CI 1.0–3.0) [5] and a Ugandan cohort study (aOR 3.14, 95%CI 0.95–10.34) [11]. Similarly, a South African study reported that drinking >20 units of alcohol per week, compared to no alcohol use, was associated with detectable viral load (aOR 7.53, 95%CI 1.04–54.55) [10].

In this cohort of Kenyan FSWs, hazardous, harmful, or dependent alcohol use was independently associated with lower self-reported adherence, but not with detectable viral load. In people taking modern ART regimens, adherence as low as 80% may be sufficient to maintain viral suppression [12], providing a potential explanation for these seemingly inconsistent findings. The low percentage of women reporting harmful alcohol use or alcohol dependency in this cohort could also partially explain these results, as previous research has suggested that non-adherence increases with increasing levels of alcohol use [3]. Alternatively, it is possible that women who are comfortable reporting heavier alcohol use are also willing to report lower ART adherence. Over half of women in this cohort were over 40 years old, and older age was associated with lower alcohol use. Exploring the association between alcohol use and viral load in a younger FSW population that has higher alcohol intake could be an avenue for future research.

This study had several strengths. The research was conducted in a population for which we have minimal data on this topic. The longitudinal design provided repeated measures of both exposure and outcome variables. This design also allowed for evaluation of temporal relationships between alcohol use and measures of viral load and ART adherence. Collecting both self-reported adherence and viral load enabled us to observe how each was associated with alcohol use within the same population. Finally, the AUDIT score provided a standardized and validated measure, allowing comparison to other studies [21].

This analysis also had a number of limitations. First, the AUDIT score is focused on 12-month recall of alcohol use. This measure was chosen because of its feasibility for implementation in HIV care settings, but does not provide information about event-level associations between alcohol use and non-adherence. Second, alcohol use and poor ART adherence are both sensitive topics, and subject to under-reporting due to social desirability bias. Third, this was an older cohort, with a median age of 42 years. While this is reflective of the aging of the HIV-positive population in Africa [43], it limits the ability of these analyses to examine the association between AUDIT score and detectable viral load in younger women. Finally, most alcohol use in this cohort was moderate, with few AUDIT scores indicating harmful alcohol use or alcohol dependency. As a result, we were not able to examine associations with these categories separately.

## Conclusions

This study adds to the limited literature on the relationship between alcohol use and HIV viral load suppression in ART-treated African FSWs. The results of this study suggest that targeted interventions to address alcohol use could impact ART adherence, but this effect may not be large enough to improve viral suppression, particularly in populations like this one, where few women reported harmful or dependent alcohol use.

## Supporting information

S1 File Sensitivity analysis. (DOCX)

S2 File CRFs. (PDF)
