# Supplementary material for: Alcohol use and viral suppression in HIV-positive Kenyan female sex workers on antiretroviral therapy
Source: PLoS One. 2020 Nov 24;15(11):e0242817. doi: 10.1371/journal.pone.0242817 (PMC7685481; doi:10.1371/journal.pone.0242817)
Supplement: S1 File — (DOCX) [file pone.0242817.s001.docx]

**Supporting Information: Sensitivity analysis**

For primary analyses, after estimating unadjusted associations, a multivariable model was built using a forward manual selection process. Sensitivity analyses, reported here, were conducted to fully fit each model with all covariates found to be associated with the outcome examined in that model. All models were adjusted for age based on consistent prior findings of associations with alcohol use and adherence. Additional covariates including PHQ-9 score, intimate partner violence, and sexual behavior, were selected as potential confounders based on published associations. These variables were modelled as time-varying in analyses. In sensitivity analysis, any variables that shifted the effect estimate for the association between AUDIT score ≥7 and the outcome by >10% was retained in a final, fully adjusted model. Table S1 below shows the result of the primary analysis, comparing the univariate model, the primary analysis model (forward step-wise selection), and the model that was fully fit with all associated covariates. For the outcomes of viral load and late refill, the fully fit model did not differ from the forward step-wise selection model. For the self-rated adherence outcomes, the effect estimate in the fully fit model was somewhat attenuated compared to the model using forward step-wise selection, but the direction, statistical significance, and inference did not differ between the two models.

**S1 Table 1.** **Comparison of univariate, forward step-wise selection multivariate, and fully fit model multivariate risk ratios (RR) and 95% confidence intervals (CI) for the association of hazardous/harmful/dependent alcohol use, as measured by AUDIT score ≥ 7, on viral suppression and self-reported and objective measures of ART adherence.**

| **Outcome** | **Univariate regression estimates** | | **Multivariate: Forward step-wise selection model** | | **Multivariate: fully fit model** | |
| --- | --- | --- | --- | --- | --- | --- |
|  | Risk Ratio (95%CI) | p-value | Adjusted Risk Ratio (95%CI) | p-value | Adjusted Risk Ratio (95%CI) | p-value |
| **Viral load** |  |  |  |  |  |  |
| Undetectable | REFERENCE |  | REFERENCE |  | REFERENCE |  |
| Detectable | 1.59 (0.93, 2.69) | 0.084 | 1.10 (0.63, 1.92)^a^ | 0.739 | 1.06 (0.57, 1.97)^b^ | 0.848 |
| **ART refill** |  |  |  |  |  |  |
| Timely | REFERENCE |  | REFERENCE |  | REFERENCE |  |
| Late (>48 hours) | 1.38 (0.99, 1.93) | 0.056 | 1.13 (0.82, 1.56) ^a^ | 0.442 | 1.14 (0.84, 1.55) ^c^ | 0.407 |
| **Self-rated ability to take ART** |  |  |  |  |  |  |
| Excellent | REFERENCE |  | REFERENCE |  | REFERENCE |  |
| Less than excellent | 3.68 (2.48, 5.44) | <0.001 | 2.41 (1.48, 3.92) ^d^ | <0.001 | 2.15 (1.25, 3.71) ^e^ | 0.006 |
| **Self-rated ART adherence** |  |  |  |  |  |  |
| Complete (100%) | REFERENCE |  | REFERENCE |  | REFERENCE |  |
| Incomplete (<100%) | 3.44 (2.44, 4.85) | <0.001 | 2.40 (1.71, 3.38) ^a^ | <0.001 | 2.30 (1.48, 3.58) ^f^ | <0.001 |

^a^Adjusted for age

^b^Adjusted for age, number of sex acts in last week, number of sex partners in last week, PHQ-9 score, CD4 count at baseline

^c^Adjusted for age, workplace, PHQ-9, number of years in sex work at baseline

^d^Adjusted for age and number of sex acts in the last week

^e^Adjusted for age, number of sex acts in last week, workplace, number of sex partners in last week, PHQ-9 score, CD4 count at baseline, number of years in sex work at baseline, interpersonal violence in the last 12 months

^f^Adjusted for age, workplace, PHQ-9, number of sex partners in last week, number of sex partners in last week, CD4 count at baseline, number of years in sex work at baseline, interpersonal violence in the last 12 months
