# Supplementary material for: Alcohol use and viral suppression in HIV-positive Kenyan female sex workers on antiretroviral therapy
Source: PLoS One. 2020 Nov 24;15(11):e0242817. doi: 10.1371/journal.pone.0242817 (PMC7685481; doi:10.1371/journal.pone.0242817)
Supplement: S2 File — (PDF) [file pone.0242817.s002.pdf]

LIFECOURSE ENROLLMENT QUESTIONNAIRE

Unless otherwise specified in a question or section text, 0=no; 1=yes

Enter visit number:	**VISIT 0**
1. Date (day/month/year)	___/___/___
2. Urine B-hCG result *0=not pregnant; 1=pregnant*	__
**In this study, we are interested in learning about the health and life experiences of women with HIV. First, I would like to ask you about antiretroviral therapy (ART).**	
3a. Are you <u>currently</u> on ART? ***(If "0=no," skip to Question 4)***	__
b. When did you start ART? (day/month/year) *(This does not include short-term ARVs for PMTCT; e.g. Plan A or Plan B)*	__/__/__
**Now I want you to think about your current or most recent regular male sex partner. This could be a husband or a partner with whom you have (or had) a committed relationship.** *Prompt:* **This is not someone with whom you have had only casual sex or sex in exchange for money or gifts. Some women may not have had a regular partner.**	
4. Do you have a current or most recent regular partner? ***(If "0=no," skip to Question 6)***	__
5. During the last three months, have you had a regular partner?	__
6. During the last three months, have you had a casual partner?	__
7. Current marital status: *0=never married; 1=currently married; 2=widowed/divorced*	__
**Next, I'd like to ask you some questions about pregnancies.**	
8. How many pregnancies have you had, including current one if pregnant? ***(If "0," skip to Question 11)***	__
9. What was the date of your last delivery? (day/month/year)	__/__/__
10. How many live births have you had? ***(Skip to Question 15 if pregnant)***	__
11. Are you trying to become pregnant?	__
12. Do you want to have any/more children?	__
13. How would your current or most recent regular partner feel if you became pregnant? *0=very excited; 1=somewhat excited; 2=would not care; 3=very upset; 4=does not have a regular or current partner*	__
14. Menopausal *(follow menopause clinical decision making tool)*:	__
**Now I would like to ask you some questions about your thoughts and moods. <u>Over the last 2 weeks</u>, how often have you been bothered by the following problems:**	
<u>Responses for questions 15-23:</u>	
0=not at all; 1=several days; 2=more than half the days; 3=nearly every day	
15. Little interest or pleasure doing things?	__
16. Feeling down, depressed, or hopeless?	__
17. Trouble falling or staying asleep, or sleeping too much?	__
18. Feeling tired or having little energy?	__
19. Poor appetite or overeating?	__
20. Feeling bad about yourself- or that you are a failure or have let yourself or your family down?	__
21. Trouble concentrating on things, such as listening to the radio?	__

22. Moving or speaking so slowly that other people could have noticed? Or the opposite- being so fidgety or restless that you have been moving around a lot more than usual? __
23. Thoughts that you would be better off dead, or of hurting yourself in some way? __

    (If "0=not at all" for all questions from 15 through 23, skip to Question 25)
24. If you had any of these problems, how difficult have these problems made it for you to do your work, take care of things at home, or get along with people? __

    0=not difficult at all;1=somewhat difficult; 2=very difficult; 3=extremely difficult

Now I'd like to ask you some questions about your use of tobacco and drugs.

25. Do you:
    a. Smoke? If YES, cigarettes per day. __
    b. Chew miraa, or khat? If YES, times per month. __
    c. Smoke marijuana? If YES, times per month. __
    d. Use cocaine? If YES, times per month. __

       If YES, how? 1=sniff; 2=smoke; 3=inject; 4=other, specify __________ __
    e. Use intravenous drugs? If YES, times per month. __

Now I'd like to ask you some questions about your use of alcoholic beverages <u>during the last year</u>.

26. How often do you have a drink containing alcohol, such as beer, wine, spirits, or locally brewed alcohol (such as changaa, mnazi, busaa, or muratina)? __

    0=never ***(skip to Question 36)****; 1=monthly or less; 2=2 to 4 times a month;3=2 to 3 times a week;4=4 or more times a week*
27. How many drinks containing alcohol do you have on a typical day when you are drinking? One 500mL bottle of beer like a Tusker counts as 1.5 drinks. A glass of wine or locally brewed alcohol counts as one drink. One 'shot' of spirits is one drink. __

    0=1 or 2; 1=3 or 4; 2=5 or 6; 3=7, 8, or 9; 4=10 or more
28. How often do you have 6 or more drinks on one occasion? __

    0=never; 1=less than monthly; 2=monthly; 3=weekly; 4=daily or almost daily

How often <u>during the last year</u> have you:

<u>Responses for questions 29-33:</u>

0=never; 1=less than monthly; 2=monthly; 3=weekly; 4=daily or almost daily

29. Found that you were not able to stop drinking once you had started? __
30. Failed to do what was normally expected from you because of drinking? __
31. Been unable to remember what happened the night before because you had been drinking? __
32. Needed an alcoholic drink first thing in the morning to get yourself going after a night of heavy drinking? __
33. Had a feeling of guilt or remorse after drinking? __
34. Have you or someone else <u>ever</u> been injured as a result of your drinking? __

    0=no; 1=yes, but not in the past year; 2=yes, during the past year

35. Has a relative, friend, doctor, or another health professional ever expressed concern about your drinking or suggested you cut down? __
0=no; 1=yes, but not in the past year; 2=yes, during the past year

Now, I would like to ask you a couple of questions about your HIV test results.

36a. Have you shared your HIV test results with someone? ***(If "0=no," skip to Question 37)*** __

b. With whom? ***Enter number of people in each category:***

__parent	*__boyfriend/partner*	*__sibling*	*__health provider outside this clinic*
__husband	*__other friend/acquaintance*	*__religious leader*	*__other (specify:____________)*
__child	*__employer*		

(If no current or most recent regular partner in Question 4, skip to Question 58)
Next, I would like to ask some questions about your current and past relationships, and how your husband/male partner treats (treated) you. Please answer these questions about the same person you identified as a current or most recent regular male sex partner earlier.
Thinking about your current or most recent regular partner, would you say it is generally true that he:

37. Tries to keep you from seeing your friends? __
38. Tries to restrict contact with your family of birth? __
39. Insists on knowing where you are at all times? __
40. Ignores you and treats you indifferently? __
41. Gets angry if you speak with another man? __
42. Is often suspicious that you are unfaithful? __
43. Expects you to ask his permission before seeking health care for yourself? __

**The next questions are about things that happen to many women and that your current or most recent regular partner may have done to you. Has your current regular or most recent regular partner:** *0=never **(skip to next question)**; 1=once; 2=few; 3=many*	**A) Ever?**	**B) In past 12 months?**
44. Insulted you or made you feel bad about yourself?	__	__
45. Belittled or humiliated you in front of other people?	__	__
46. Done things to scare or intimidate you on purpose, e.g. by the way he looked at you, by yelling or smashing things?	__	__
47. Threatened to hurt you or someone you care about?	__	__
48. Slapped you or thrown something at you that could hurt you?	__	__
49. Pushed or shoved you or pulled your hair?	__	__
50. Hit you with his fist or with something else that could hurt?	__	__
51. Kicked you, dragged you, or beaten you up?	__	__
52. Choked or burnt you on purpose?	__	__
53. Threatened to use or actually used a gun, knife, or other weapon against you?	__	__
54. Physically forced you to have sexual intercourse when you did not want to?	__	__
55. Did you have sexual intercourse you did not want to because you were afraid of what he might do?	__	__
56. Did he force you to do something sexual that you found degrading or humiliating?	__	__

(If responses to all Questions 44 – 56 are "0=never," skip to Question 58)

57. Have you been injured as a result of these acts by your current regular partner? __ __

Please think of the acts that we talked about before.

Since the age of 15 years has anyone (other than your current or most recent regular partner):

Responses for questions 58 and 59:

0=never; 1=once or twice; 2=a few times; 3=many times

58. Beaten or physically mistreated you in any way? __ __
59. Forced you to have sex or perform a sexual act when you did not want to? __ __

(If responses to both 58 AND 59 are "0=never," skip to Question 61)

60. Who did this to you? ***Enter 0=no or 1=yes for each response:***

__father/stepfather	*__religious leader*	*__someone at work*	*__boyfriend*
__other male relative	*__police*	*__neighbor/acquaintance*	*__client*
__female relative	*__stranger*	*__teacher*	*__other, specify:____________*

61a. Have you sought help for any violence that you experienced? __

(If "0=no," skip to Question 62)

b. Specify where: ____

62a. Have you ever had sex in exchange for money or gifts? __

b. In the last 12 months, have you had sex in exchange for money or gifts? __

COMMENTS:____

____

____

Initials of person completing questionnaire: __
Initials of person entering data in computer: __
Initials of person performing line listing: __

PHYSICAL EXAMINATION

Unless otherwise specified, 0=no, 1=yes

	VISIT ____	VISIT ____	VISIT ____	VISIT ____	VISIT ____	VISIT ____	VISIT ____
1. Date *(day/month/year)*	___/___/___	___/___/___	___/___/___	___/___/___	___/___/___	___/___/___	___/___/___
**GENERAL EXAMINATION**							
2. Temperature axillary *(in degrees Celsius)*	__ __.__	__ __.__	__ __.__	__ __.__	__ __.__	__ __.__	__ __.__
3. Weight *(kg)*	__ __ __	__ __ __	__ __ __	__ __ __	__ __ __	__ __ __	__ __ __
4. Height *(cm)*	__ __ __	__ __ __	__ __ __	__ __ __	__ __ __	__ __ __	__ __ __
5. Karnofsky Performance score (see posted definitions)	__	__	__	__	__	__	__
**PELVIC EXAMINATION**							
6. Circumcision *(0=no; 1=Sunna; 2=intermediate; 3=Pharaonic; 4=infibulation)*	__	__	__	__	__	__	__
7. Vulvitis	__	__	__	__	__	__	__
8. Vulvar ulcer	__	__	__	__	__	__	__
9. Vulvar warts	__	__	__	__	__	__	__
10. Vaginal ulcer	__	__	__	__	__	__	__
11. Vaginal warts	__	__	__	__	__	__	__
12. Vaginal discharge							
a. Amount *(0=none; 1=small; 2=moderate; 3=profuse; 4=menses)*	__	__	__	__	__	__	__
b. Color *(1=white/clear; 2=yellow/gray; 3=brown; 4=blood)*	__	__	__	__	__	__	__
c. Character *(1=non-homogenous (normal); 2=homogenous; 3=plaques; 4=frothy)*	__	__	__	__	__	__	__
13. Vaginal pH	__	__	__	__	__	__	__
14. Whiff test	__	__	__	__	__	__	__
15. Cervical ulcer	__	__	__	__	__	__	__
16. Cervical warts	__	__	__	__	__	__	__
17. Cervical mucopus	__	__	__	__	__	__	__
18. Cervical ectopy (%) – naked eye	__	__	__	__	__	__	__
19. Uterine/adnexal tenderness	__	__	__	__	__	__	__
20. Pregnancy *(0=no; gestational age 1-9=yes)*	__	__	__	__	__	__	__

**DIAGNOSES**	**VISIT ____**	**VISIT ____**	**VISIT ____**	**VISIT ____**	**VISIT ____**	**VISIT ____**	**VISIT ____**
21. HIV clinical staging *(1 – 4)*	__	__	__	__	__	__	__
22. List clinical conditions seen in patient, corresponding with current HIV clinical stage (use numbering of "HIV Clinical Staging" sheet)							
a. Variable	__ __	__ __	__ __	__ __	__ __	__ __	__ __
b. Variable	__ __	__ __	__ __	__ __	__ __	__ __	__ __
c. Variable	__	__	__	__	__	__	__
d. Variable	__ __	__ __	__ __	__ __	__ __	__ __	__ __
e. Variable	__ __	__ __	__ __	__ __	__ __	__ __	__ __

COMMENTS:____

____

____

____

____

____

____

Initials of person completing questionnaire:	__	__	__	__	__	__	__
Initials of person entering data in computer:	__	__	__	__	__	__	__
Initials of person performing line listing:	__	__	__	__	__	__	__

LABORATORY RESULTS

Unless otherwise specified, 0=negative, 1=positive

		VISIT ____	VISIT ____	VISIT ____	VISIT ____	VISIT ____	VISIT ____	VISIT ____
1.	Date *(day/month/year)*	___/___/__	___/___/__	___/___/__	___/___/__	___/___/__	___/___/__	___/___/__
2.	Trichomonas wet prep	__	__	__	__	__	__	__
3.	Vaginal Candida *(KOH)*	__	__	__	__	__	__	__
4.	Cervical gram stain (0-30; if >30=31)	__ __	__ __	__ __	__ __	__ __	__ __	__ __
5.	BV gram stain, score 1-10	__ __	__ __	__ __	__ __	__ __	__ __	__ __
6.	Gonorrhea culture	__	__	__	__	__	__	__
7.	Cervical gram stain *(GC)*	__	__	__	__	__	__	__
8.	Aptima gonorrhea test (0=neg,1=pos, 7=weak pos)	__	__	__	__	__	__	__
9.	Aptima Chlamydia test (0=neg,1=pos,7=weak pos)	__	__	__	__	__	__	__
10.	Aptima *Trichomonas* test (0=neg,1=pos,7=weak pos)	__	__	__	__	__	__	__
11.	RPR *(if >32=33)*	__ __	__ __	__ __	__ __	__ __	__ __	__ __
12.	TPHA *(0-4)*	__	__	__	__	__	__	__
13.	CD4+ lymphocytes *(cells/mm^3)*	__ __ __ __	__ __ __ __	__ __ __ __	__ __ __ __	__ __ __ __	__ __ __ __	__ __ __ __
14.	CD8+ lymphocytes *(cells/mm^3)*	__ __ __ __	__ __ __ __	__ __ __ __	__ __ __ __	__ __ __ __	__ __ __ __	__ __ __ __
15.	Sputum smear AFB 1	__	__	__	__	__	__	__
16.	Sputum smear AFB 2	__	__	__	__	__	__	__
17.	Sputum smear AFB 3	__	__	__	__	__	__	__
18.	Chest X-ray	__	__	__	__	__	__	__
19.	Pleural effusion	__	__	__	__	__	__	__
20.	Infiltrate *(0=no; 1=yes)*	__	__	__	__	__	__	__
21.	Cavitation	__	__	__	__	__	__	__
22.	Plasma drug level *(µg/mL)*	__ __ __ __	__ __ __ __	__ __ __ __	__ __ __ __	__ __ __ __	__ __ __ __	__ __ __ __

COMMENTS:____

____

____

Initials of person completing form:	______	______	______	______	______	______	______
Initials of person entering data into computer:	______	______	______	______	______	______	______
Initials of person performing line listing:	______	______	______	______	______	______	______

LIFECOURSE GENERAL TREATMENT QUESTIONNAIRE

Unless otherwise specified, 0=no, 1=yes	VISIT _____	VISIT _____	VISIT _____	VISIT _____	VISIT _____	VISIT _____	VISIT _____
1. Date ((day/month/year)	___/___/____	___/___/____	___/___/____	___/___/____	___/___/____	___/___/____	___/___/____
2. Examination done?	__	__	__	__	__	__	__
3a. Any medication dispensed?	__	__	__	__	__	__	__
b. Treatment for findings on visit date:	_____________	_____________	_____________	_____________	_____________	_____________	_____________
**LONG-TERM MEDICATIONS**							
4. Taking antiretrovirals?	__	__	__	__	__	__	__
5. Taking septrin prophylaxis?	__	__	__	__	__	__	__
6. Taking isoniazid preventive therapy?	__	__	__	__	__	__	__
7. Taking treatment for active TB?	__	__	__	__	__	__	__

COMMENTS:____
____
____
____

Initials of person completing questionnaire:	__	__	__	__	__	__	__
Initials of person entering data in computer:	__	__	__	__	__	__	__
Initials of person performing line listing:	__	__	__	__	__	__	__

LIFECOURSE GENERAL MONTHLY FOLLOW UP QUESTIONNAIRE

Unless otherwise specified in a question or section text, 0=no, 1=yes

Enter visit number:	**VISIT___**	**VISIT___**	**VISIT___**	**VISIT___**	**VISIT___**	**VISIT___**	**VISIT___**
1. Date (day/month/year)	___/___/___	___/___/___	___/___/___	___/___/___	___/___/___	___/___/___	___/___/___
2. Current method of contraception:	__	__	__	__	__	__	__
0=none; 1=condoms only; 2=OCP; 3=spermicides;							
4=diaphragm; 5=Depo provera; 6=IUD; 7=tubal ligation;							
8=hysterectomy; 9=Norplant; 10=other, specify _________							
3. Date of last menstrual period (day/month/year)	__/__/__	__/__/__	__/__/__	__/__/__	__/__/__	__/__/__	__/__/__
4. Do you have any serious medical problems?	__	__	__	__	__	__	__
Describe ____							
**<u>During the last week:</u>**							
5. # of different sex partners	__	__	__	__	__	__	__
6. Frequency of vaginal intercourse	__	__	__	__	__	__	__
7. Frequency of vaginal intercourse with condoms	__	__	__	__	__	__	__
8. Frequency of anal intercourse	__	__	__	__	__	__	__
9. Frequency of anal intercourse with condoms	__	__	__	__	__	__	__
**<u>In the last month:</u>**							
10a. Have you had any new sex partners?	__	__	__	__	__	__	__
b. If yes, how many?	__	__	__	__	__	__	__
**<u>General symptoms:</u>**							
11. Fever, constant or intermittent >1month?	__	__	__	__	__	__	__
12. Weight loss	__	__	__	__	__	__	__
13. Night sweats	__	__	__	__	__	__	__
14. Loss of appetite	__	__	__	__	__	__	__
15. Vaginal itching/burning	__	__	__	__	__	__	__
16. Vaginal discharge	__	__	__	__	__	__	__

Enter visit number:	VISIT___	VISIT___	VISIT___	VISIT___	VISIT___	VISIT___	VISIT___
17. Lower abdominal pain	__	__	__	__	__	__	__
18a. Genital sores	__	__	__	__	__	__	__
b. If yes, have they persisted for >1 month?	__	__	__	__	__	__	__
**Skin:**							
19. Itchy skin rash	__	__	__	__	__	__	__
20. Sores in mouth and/or lips for >1 month	__	__	__	__	__	__	__
21. Shingles	__	__	__	__	__	__	__
22a. Other skin conditions (fungus, scabies,…)	__	__	__	__	__	__	__
b. Specify ___________							
**Gastro-intestinal:**							
23. Retrosternal pain when swallowing	__	__	__	__	__	__	__
24. Diarrhea, constant or intermittent for >1 month (≥3 loose stools/day)	__	__	__	__	__	__	__
25. Abdominal discomfort	__	__	__	__	__	__	__
26. Nausea	__	__	__	__	__	__	__
**Neurologic:**							
27. Pain, numbness, or tingling in your hands or feet?	__	__	__	__	__	__	__
**Chest:**							
28. Cough, acute, with or without sputum (<1 month)	__	__	__	__	__	__	__
29. Cough, constant or intermittent for >1 month	__	__	__	__	__	__	__
30. Dyspnea	__	__	__	__	__	__	__
31. Pleuritic chest pain	__	__	__	__	__	__	__
32. Palpitations (extra heart beats)	__	__	__	__	__	__	__
33a. Do/did you receive treatment for tuberculosis?	__	__	__	__	__	__	__
b. If yes, which clinic gives/gave you anti-TB drugs?	________	________	________	________	________	________	________

Enter visit number:	**VISIT___**	**VISIT___**	**VISIT___**	**VISIT___**	**VISIT___**	**VISIT___**	**VISIT___**
**Since last visit:**							
34a. Have you visited any other health care provider or facility?	__	__	__	__	__	__	__
If no, STOP here. If yes, complete 34b – d.							
b. If yes, which provider or facility?	________	________	________	________	________	________	________
a. If yes, did you receive any kind of treatment?	________	________	________	________	________	________	________
b. If yes, what treatment? (e.g. name of medicine)	________	________	________	________	________	________	________

COMMENTS:____

____

____

____

Initials of person completing questionnaire:	__	__	__	__	__	__	__
Initials of person entering data in computer:	__	__	__	__	__	__	__
Initials of person performing line listing:	__	__	__	__	__	__	__

Study ID # __ __ __ __ __ __ __ __

ANTIRETROVIRAL VISIT QUESTIONNAIRE

**Unless otherwise specified, 0=no, 1=yes.**	**VISIT __**	**VISIT__**	**VISIT __**	**VISIT __**	**VISIT__**	**VISIT __**	**VISIT__**
1. Date (day/month/year)	___/___/___	___/___/___	___/___/___	___/___/___	___/___/___	___/___/___	___/___/___
ADHERENCE TO TREATMENT<br>*(To be filled out by nurse.)*							
2. Rate your ability to take your medications as prescribed during the past month: 0=very poor; 1=poor; 2=fair; 3=good; 4=very good; 5=excellent	____	____	____	____	____	____	____
3. Brought mediset to visit?<br>*If no, skip to question 5.*	____	____	____	____	____	____	____

4. If yes, doses taken by pill count
For each drug, enter number of pills dispensed last visit minus number left in mediset. Then skip to question 6.

Lamivudine	___ ___	___ ___	___ ___	___ ___	___ ___	___ ___	___ ___
Stavudine	___ ___	___ ___	___ ___	___ ___	___ ___	___ ___	___ ___
Nevirapine	___ ___	___ ___	___ ___	___ ___	___ ___	___ ___	___ ___
Zidovudine	___ ___	___ ___	___ ___	___ ___	___ ___	___ ___	___ ___
Didanosine	___ ___	___ ___	___ ___	___ ___	___ ___	___ ___	___ ___
Lopinavir/Ritonavir	___ ___	___ ___	___ ___	___ ___	___ ___	___ ___	___ ___
Abacavir	___ ___	___ ___	___ ___	___ ___	___ ___	___ ___	___ ___
Efavirenz, 600-mg	___ ___	___ ___	___ ___	___ ___	___ ___	___ ___	___ ___
Efavirenz, 200-mg	___ ___	___ ___	___ ___	___ ___	___ ___	___ ___	___ ___
Tenofovir	___ ___	___ ___	___ ___	___ ___	___ ___	___ ___	___ ___

**VISIT NUMBER**	_	_	_	_	_	_	_
5. Doses taken by patient recall<br>*For each drug, enter number of pills dispensed last visit minus number left in mediset (by patient recall).*							
Lamivudine	___ ___	___ ___	___ ___	___ ___	___ ___	___ ___	___ ___
Stavudine	___ ___	___ ___	___ ___	___ ___	___ ___	___ ___	___ ___
Nevirapine	___ ___	___ ___	___ ___	___ ___	___ ___	___ ___	___ ___
Zidovudine	___ ___	___ ___	___ ___	___ ___	___ ___	___ ___	___ ___
Didanosine	___ ___	___ ___	___ ___	___ ___	___ ___	___ ___	___ ___
Lopinavir/Ritonavir	___ ___	___ ___	___ ___	___ ___	___ ___	___ ___	___ ___
Abacavir	___ ___	___ ___	___ ___	___ ___	___ ___	___ ___	___ ___
Efavirenz, 600-mg	___ ___	___ ___	___ ___	___ ___	___ ___	___ ___	___ ___
Efavirenz, 200-mg	___ ___	___ ___	___ ___	___ ___	___ ___	___ ___	___ ___
Tenofovir	___ ___	___ ___	___ ___	___ ___	___ ___	___ ___	___ ___
6. Visual Analog Scale (in cm)<br>*Measure on VAS sheet and enter here.*	___ ___ . ___	___ ___ . ___	___ ___ . ___	___ ___ . ___	___ ___ . ___	___ ___ . ___	___ ___ . ___
7. Has received DAART in last week?	_____	_____	_____	_____	_____	_____	_____
8. Refilled for how many doses?<br>*Enter total number of doses sent out with patient. Give 64 doses at each visit unless patient requests more.*	___ ___	___ ___	___ ___	___ ___	___ ___	___ ___	___ ___
9. Pills dispensed							
Lamivudine (standard = 64)	___ ___	___ ___	___ ___	___ ___	___ ___	___ ___	___ ___
Stavudine (standard = 64)	___ ___	___ ___	___ ___	___ ___	___ ___	___ ___	___ ___
Nevirapine (standard = 64)	___ ___	___ ___	___ ___	___ ___	___ ___	___ ___	___ ___
Zidovudine (standard = 64)	___ ___	___ ___	___ ___	___ ___	___ ___	___ ___	___ ___
Didanosine (standard = 128)	___ ___	___ ___	___ ___	___ ___	___ ___	___ ___	___ ___
Lopinavir/Ritonavir (standard = 192)	___ ___	___ ___	___ ___	___ ___	___ ___	___ ___	___ ___
Abacavir (standard = 64)	___ ___	___ ___	___ ___	___ ___	___ ___	___ ___	___ ___
Efavirenz, 600-mg (standard = 32)	___ ___	___ ___	___ ___	___ ___	___ ___	___ ___	___ ___
Efavirenz, 200-mg (standard = 32)	___ ___	___ ___	___ ___	___ ___	___ ___	___ ___	___ ___
Tenofovir (standard = 32)	___ ___	___ ___	___ ___	___ ___	___ ___	___ ___	___ ___

Study ID # __ __ __ __ __ __ __ __

**VISIT NUMBER**	_	_	_	_	_	_	_
ARV REGIMEN CHANGES							
10. ARV regimen changed or discontinued?<br>0 = No, 1 = Changed, 2 = Discontinued<br>*If no, stop here.*	_____	_____	_____	_____	_____	_____	_____
11. *If changed or discontinued, enter reasons (0=no, 1=yes).*							
11a. Hepatitis, asymptomatic	_____	_____	_____	_____	_____	_____	_____
11b. Hepatitis, symptomatic	_____	_____	_____	_____	_____	_____	_____
11c. Severe rash	_____	_____	_____	_____	_____	_____	_____
11d. Treatment failure	_____	_____	_____	_____	_____	_____	_____
11e. Patient preference	_____	_____	_____	_____	_____	_____	_____
11f. Pregnancy	_____	_____	_____	_____	_____	_____	_____
11g. Other *(describe in comments)*	_____	_____	_____	_____	_____	_____	_____
Initials of person completing interview	_____	_____	_____	_____	_____	_____	_____
Initials of person entering data in computer	_____	_____	_____	_____	_____	_____	_____
Initials of person performing line listing	_____	_____	_____	_____	_____	_____	_____

COMMENTS: *(comments should be dated and signed with your initials)*

Visual Analog Scale for Adherence to Antiretrovirals

Put a mark on the line below at the point showing your best estimate of how much medication you have taken in the last month. We would be surprised if this was 100% for most people.

Examples: 0% means you have taken no medication
50% means you have taken half your medication
100% means you have taken every single dose

0 10 20 30 40 50 60 70 80 90 100

ID: ___ ___ ___ ___ ___

Date: ___ ___ / ___ ___ / ___ ___

Distance from 100 in cm: ___ ___ . ___

Version Oct 17 2013

LIFECOURSE QUARTERLY QUESTIONNAIRE

Unless otherwise specified in a question or section text, 0=no; 1=yes

		VISIT___	VISIT___	VISIT___	VISIT___
	Enter visit number:				
1.	Date (day/month/year)	___/___/___	___/___/___	___/___/___	___/___/___
	**I want you to think about your current or most recent regular male sex partner. This could be a husband or a partner with whom you have (or had) a committed relationship.** *Prompt:* **This is not someone with whom you have had only casual sex or sex in exchange for money or gifts. Some women may not have had a regular partner.**				
2.	During the last three months, have you had a regular partner?	__	__	__	__
3.	During the last three months, have you had a casual partner?	__	__	__	__
2.	Urine B-hCG result 0=not pregnant; 1=pregnant ***(STOP)***	__	__	__	__
	**Now I'd like to ask you some questions about becoming pregnant.**				
3.	Are you trying to become pregnant?	__	__	__	__
4.	Do you want to have any/more children?	__	__	__	__

COMMENTS:____

____

____

____

____

Initials of person completing questionnaire:	__	__	__	__
Initials of person entering data in computer:	__	__	__	__
Initials of person performing line listing:	__	__	__	__

LIFECOURSE 6-MONTHLY QUESTIONNAIRE

Unless otherwise specified in a question or section text, 0=no, 1=yes

Enter visit number:	**VISIT___**	**VISIT___**
1. Date (day/month/year)	___/___/___	___/___/___
**I would like to ask you some questions about your thoughts and moods. Over <u>the last 2 weeks</u>, how often have you been bothered by the following problems:**		
<u>Responses for questions 2-10:</u>		
0=not at all; 1=several days; 2=more than half the days; 3=nearly every day		
2. Little interest or pleasure doing things?	__	__
3. Feeling down, depressed, or hopeless?	__	__
4. Trouble falling or staying asleep, or sleeping too much?	__	__
5. Feeling tired or having little energy?	__	__
6. Poor appetite or overeating?	__	__
7. Feeling bad about yourself- or that you are a failure or have let yourself or your family down?	__	__
8. Trouble concentrating on things, such as listening to the radio?	__	__
9. Moving or speaking so slowly that other people could have noticed? Or the opposite- being so fidgety or restless that you have been moving around a lot more than usual?	__	__
10. Thoughts that you would be better off dead, or of hurting yourself in some way? ***(If "0=not at all" for all questions from 2-10, skip to Question 12)***	__	__
11. If you had any of these problems, how difficult have these problems made it for you to do your work, take care of things at home, or get along with people? *0=not difficult at all; 1=somewhat difficult; 2=very difficult; 3=extremely difficult*	__	__
**Now I would like to ask you about your HIV test results.**		
12. Have you shared your HIV test results with someone? ***(If no, STOP)***	__	__

13. With whom? ***Enter number of people in each category:***

**VISIT___**		**VISIT___**	
__parent	*__boyfriend/partner*	*__parent*	*__boyfriend/partner*
__husband	*__other friend/acquaintance*	*__husband*	*__other friend/acquaintance*
__child	*__religious leader*	*__child*	*__religious leader*
__sibling	*__health provider outside this clinic*	*__sibling*	*__health provider outside this clinic*
__employer	*__other (specify:_____________)*	*__employer*	*__other (specify:_____________)*

COMMENTS:____

____

____

Initials of person completing questionnaire:	__	__
Initials of person entering data in computer:	__	__
Initials of person performing line listing:	__	__

LC_6MO.doc – version 2012-10-09

LIFECOURSE ANNUAL QUESTIONNAIRE

Unless otherwise specified in a question or section text, 0=no, 1=yes

		VISIT___
	Enter visit number:	
1.	Date (day/month/year):	___/___/___
2.	Current marital status:<br>*0=never married; 1=currently married; 2=widowed/divorced*	__
3.	Menopausal *(follow menopause clinical decision making tool):*	__
4a.	Have you had a delivery (including still births) in the past year? ***(If "0=no," skip to Question 5)*****:**	__
	b. What was the date of this delivery?	___/___/___

I want you to think about your current or most recent regular male sex partner <u>during the past 12 months</u>. This could be a husband or a partner with whom you have (or had) a committed relationship.
Prompt: **This is not someone with whom you have had only casual sex or sex in exchange for money or gifts. Some women may not have had a regular partner.**

5.	Do you have a current regular partner, or have you been with your most recent regular partner in the past 12 months? ***(If "0=no," skip to Question 7):***	__
6.	During the past three months, have you had a regular partner?	__
7.	During the past three months, have you had a casual partner?	__
8.	How would your current or most recent regular partner feel if you became pregnant?<br>*0=very excited; 1=somewhat excited; 2=would not care; 3=very upset; 4=does not have a regular or current partner*	__

I'd like to ask you some questions about your use of alcoholic beverages <u>during the last year</u>.

9.	How often do you have a drink containing alcohol, such as beer, wine, spirits, or locally brewed alcohol (such as changaa, mnazi, busaa, or muratina)?<br>*0=never* ***(skip to Question 19)****; 1=monthly or less; 2=2 to 4 times a month; 3=2 to 3 times a week;4=4 or more times a week*	__
10.	How many drinks containing alcohol do you have on a typical day when you are drinking? One 500mL bottle of beer like a Tusker counts as 1.5 drinks. A glass of wine or a locally brewed alcohol counts as one drink. One shot of spirits is one drink.<br>*0=1 or 2; 1=3 or 4; 2=5 or 6; 3=7, 8, or 9; 4=10 or more*	__
11.	How often do you have 6 or more drinks on one occasion?<br>*0=never; 1=less than monthly; 2=monthly; 3=weekly; 4=daily or almost daily*	__

How often <u>during the last year</u> have you:

<u>Responses for questions 12-16:</u>

0=never; 1=less than monthly; 2=monthly; 3=weekly; 4=daily or almost daily

12.	Found that you were not able to stop drinking once you had started?	__
13.	Failed to do what was normally expected from you because of drinking?	__
14.	Been unable to remember what happened the night before because you had been drinking?	__

15. Needed an alcoholic drink first thing in the morning to get yourself going after a night of heavy drinking? __
16. Had a feeling of guilt or remorse after drinking? __
17. <u>In the past year</u>, were you or someone else injured as a result of your drinking? __
    0=no; 2=yes, during the past year (Responses 0 &2 follow same format as enrollment)
18. <u>In the past year</u>, has a relative, friend, doctor, or another health professional expressed concern about your drinking or suggested you cut down? __
    0=no; 2=yes, during the past year (Responses 0 &2 follow same format as enrollment)

Now I'd like to ask you some questions about your use of tobacco and drugs.

19. Do you:
    a. Smoke? If YES, cigarettes per day. __
    b. Chew miraa, or khat? If YES, times per month. __
    c. Smoke marijuana? If YES, times per month. __
    d. Use cocaine? If YES, times per month. __
       If YES, how? *1=sniff; 2=smoke; 3=inject; 4=other, specify* _____________ __
    e. Use intravenous drugs? If YES, times per month. __

(If "0=no" current or most recent regular partner in Question 5, skip to Question 42)
Next, I would like to ask some questions about your relationships during the past 12 months, and how your husband/male partner treats (treated) you. Please answer these questions about the same person you identified as your regular male sex partner <u>during the past 12 months</u>.
Thinking about your regular partner, would you say it is generally true that he:

20. Tries to keep you from seeing your friends? __
21. Tries to restrict contact with your family of birth? __
22. Insists on knowing where you are at all times? __
23. Ignores you and treats you indifferently? __
24. Gets angry if you speak with another man? __
25. Is often suspicious that you are unfaithful? __
26. Expects you to ask his permission before seeking health care for yourself? __

The next questions are about things that happen to many women and that a regular partner may have done to you. <u>In the past 12 months</u>, has your regular partner:
0=never; 1=once; 2=few; 3=many;

27. Insulted you or made you feel bad about yourself? __
28. Belittled or humiliated you in front of other people? __
29. Done things to scare or intimidate you on purpose, e.g. by the way he looked at you, by yelling or smashing things? __
30. Threatened to hurt you or someone you care about? __
31. Slapped you or thrown something at you that could hurt you? __
32. Pushed or shoved you or pulled your hair? __
33. Hit you with his fist or with something else that could hurt? __
34. Kicked you, dragged you, or beaten you up? __
35. Choked or burnt you on purpose? __

36. Threatened to use or actually used a gun, knife, or other weapon against you? __
37. Physically forced you to have sexual intercourse when you did not want to? __
38. Did you have sexual intercourse you did not want to because you were afraid of what he might do? __
39. Did he force you to do something sexual that you found degrading or humiliating? __

(If responses to all Questions 27-39 are "0=never," skip to Question 41)

40. Have you been injured as a result of these acts by your current regular partner? Please think of the acts that we talked about before. __

In the past 12 months, has anyone (other than your regular partner):

Responses for questions 41 and 42:

0=never; 1=once or twice; 2=a few times; 3=many times

41. Beaten or physically mistreated you in any way? __
42. Forced you to have sex or perform a sexual act when you did not want to? __

(If responses to both 41 AND 42 are "0=never," skip to Question 44)

43. Who did this to you? *Enter 0=no or 1=yes for each response:*

__father/stepfather	*__religious leader*	*__someone at work*	*__boyfriend*
__other male relative	*__police*	*__neighbor/acquaintance*	*__client*
__female relative	*__stranger*	*__teacher*	*__other, specify:____________*

44a. Have you sought help for any violence that you experienced? __

(If "0=no," skip to Question 45)

b. Specify where: ____

45. In the past 12 months, have you had sex in exchange for money or gifts? __

COMMENTS:____

____

____

____

____

Initials of person completing questionnaire: __
Initials of person entering data in computer: __
Initials of person performing line listing: __
